# Prospective Study Showing Results of Large-Diameter Femoral Heads After Cementless Total Hip Replacement

**DOI:** 10.7759/cureus.12610

**Published:** 2021-01-10

**Authors:** Gur Aziz Singh Sidhu, Harjot Kaur, Hakam Singh, Jamie Hind, Neil Ashwood

**Affiliations:** 1 Trauma and Orthopaedics, University Hospitals of Derby and Burton, Burton, GBR; 2 Trauma and Orthopaedics, Dayanand Medical College and Hospital, Ludhiana, IND; 3 Anesthesia, Dayanand Medical College and Hospital, Ludhiana, IND

**Keywords:** total hip replacement (thr), harris hip score (hhs), metal on polyethylene, large-diameter femoral head

## Abstract

Introduction

Large-diameter femoral heads (≥36 mm) were introduced to decrease instability and improve the range of motion of the hip. We hypothesized regarding the clinical outcome and complications (dislocation, implant survivorship, and functional scores) following total hip replacement (THR) surgery in an Indian population who have smaller acetabulum compared to the western population.

Methodology

A prospective study was conducted at a tertiary hospital from November 2011 to July 2013. A total of 70 patients with hip pathology were operated by a senior surgeon for THR using the anterolateral approach. The Harris Hip Scores were recorded pre and postoperatively in all patients. Postoperatively, radiographs were taken to check for evidence of implant loosening or osteolysis. The patients were followed up till a mean follow-up of 86.52 months (range: 74 to 108 months) in our cohort. Detailed clinical and radiographic results were available for 59 patients, while six died (three died of myocardial infarction (MI), two had cerebrovascular accident (CVA), and one patient died of pulmonary embolism) and five patients were lost to follow-up.

Results

Of the 59 hips, majority (76%) had acetabular inclination of 46-55 degrees. Ninety percent of the stems were in the central position and 10% were in the varus position. The average preoperative Harris Hip Score was 38.8 ± 5.7 (range: 24-46), which increased to 90.4 ± 7.3 (range: 78-94) at the last follow-up. A total of six patients died (four died of MI and two of CVA) and two patients had infection which was treated with antibiotics. Three cases of dislocation were observed; one following a fall one year after surgery and revision total hip arthroplasty was done and two cases while getting up from the bed which were managed with closed reduction and abduction brace for six weeks. Two cases of periprosthetic fracture were observed which were managed with plating.

Conclusion

Lower dislocation rate and better range of movement reinforces the advantage of large-diameter femoral head during THR in the Indian population.

## Introduction

Total hip replacement (THR) has been recently referred to as the surgery of the century, and with the advent of improved prosthetic design and developments in bearing surfaces and materials, more is expected by surgeons and patients [1]. Hip instability leading to dislocation is a common cause for revision THR [2,3]. According to the literature, the incidence of dislocation in primary THR ranges from 0.4% to 5.8%, which increases to 20% after revision surgery [2-4].

Large-diameter femoral heads were introduced to decrease instability and improve the range of motion of the hip. The major benefit of using such femoral heads is the jump distance, the distance needed to travel farther before subluxation/dislocation occurs. In addition, such heads offer increased range of motion and prevent early component-to-component impingement. Recent advances in THR, highly cross-linked polyethylene and fourth-generation ceramic-bearing surfaces, provide benefits of large-diameter articulations with improved stability while purportedly being wear-resistant [5-7].

Despite these benefits, such heads might impinge against the anterior hip capsule or iliopsoas muscle, leading to an increased incidence of groin pain. Moreover, the incidence of groin pain ranges from 0.4% to 18% following THR [8,9]. Little has been published regarding the functional outcomes of large-diameter heads in the Indian population; hence, this study was conducted to evaluate the clinical outcome and complications (dislocation, implant survivorship, and functional scores) as well as the radio­graphic results of large-diameter THR in the Indian population.

## Materials and methods

This was a prospective study of patients undergoing THR surgery at a tertiary hospital from 2011 to 2013 after obtaining written consent and ethics clearance from the Institutional Review Board. A total of 70 patients with hip pathology were operated by a senior surgeon for THR using the anterolateral approach. The components of the Triology Acetabular Hip System (Zimmer Biomet, Warsaw, Indiana, USA) were used for implantation of the acetabulum and femur in our cohort. Preoperatively, demographic data including age, sex, weight, height, body mass index (BMI), diagnosis, and presence of any comorbidities were recorded (Table 1). In addition, the Harris Hip Scores (HHSs) of all patients were recorded preoperatively and postoperatively during subsequent follow-ups. Postoperatively, radiographs were taken to check for evidence of implant loosening or osteolysis around it. The patients were reviewed for suture removal at two weeks and then followed up at six weeks, three months, six months, and then on yearly.

The average follow-up period in our cohort ranged from 74 to 108 months (mean: 86.52 months). The detailed clinical and radiographic results are available for 59 patients, while six patients died (three died of myocardial infarction (MI), two patients had cerebrovascular accident (CVA), and one patient died of pulmonary embolism (PE)) and five were lost to follow-up.

**Table 1:** Demographic data of the study population HHS, Harris Hip Score; OA, osteoarthritis

Total patients	70 patients (70 hips)
Male/female	40 males/30 females
Age (years)	62.38 years (40-80 years)
Primary OA	3 patients
Secondary OA	67 patients
Approach	Anterolateral approach
Follow-up	86.52 months (74-108 months)
HHS (Preoperative)	38.89 (30-60)
HHS (Postoperative)	90.42 (78-94)

## Results

A single surgeon performed all the THRs in 59 patients (42 males, 17 females) using large-diameter metal heads on polyethylene liner. The mean age was 62.38 years (range: 40-80 years). The average weight of patients was 62.4 kg (range: 48.0-87.5 kg); although males weighed more than females, the difference was not statistically significant. Similarly, the average height was 162.3 cm (range: 142.3-180.6 cm) and the difference in males and females was not statistically significant. The average BMI of patients was 22.3 kg/m^2^ (range: 18.8-26.1 kg/m^2^). The duration of the surgery ranged from 54 to 90 minutes (mean: 60.2 minutes), and the average blood loss was 360 mL (280-550 mL).

The mean diameter of metal head in our cohort was 36.8 mm (range: 36-40 mm), and the mean size of acetabular implant was 55.22 mm (range: 44-58 mm). The size of the femoral stems used in our study ranged from size nine to 14 (mean: 12.2). Of the 59 hip replacements, majority (76%) had acetabular inclination of 46-55 degrees, 12 hips had inclination of 36-45 degrees, and two hips had inclination of more than 56 degrees. Ninety percent of the stems were in the central position and 10% were in the varus position. None of the cases had valgus position of the stem. The average preoperative HHS was 38.8 ± 5.7 (range: 24-46) which increased to 90.4 ± 7.3 (range: 78-94) at the last follow-up (Table 2). Six patients died (three died of MI, two patients had CVA, and one died of PE), and three deaths were observed within two years post hip surgery. Two cases of superficial infection were managed with antibiotics and strict glycemic control which were attributed to uncontrolled diabetic status postoperatively. Three cases of dislocation were observed; one following a fall one year after surgery and revision THR and two cases while getting up from the bed which were managed conservatively with closed reduction and abduction brace for six weeks. Two cases of periprosthetic fracture were observed and managed with plating.

## Discussion

Despite improvements in prosthetic design and developments in bearing surfaces/materials, dislocation is still a major issue in THR [2-4]. Several determinants responsible for dislocation of hip prosthesis have been identified, of which the most important is the diameter of the femoral head [10-14]. Large-diameter femoral heads were introduced to decrease instability and improve the range of motion of the hip [15-20].

Due to paucity of studies on large-diameter THR in the Indian population, we conducted this study. Our results are quite promising and comparable with published studies. In a randomized control trial conducted by Howie et al., significantly lower rates of dislocation were found after primary total hip arthroplasty with 36-mm heads compared with 28-mm heads (1.3% vs. 5.4%; P = 0.012) [15]. Similarly, a study by Malkani et al. found a decrease in the dislocation rate from 4.21% to 2.14%, with the use of large-diameter (>36 mm) femoral heads [16]. Conroy et al. observed similar results in the Australian National Joint Replacement Registry and reported a significant decrease in the risk for dislocation with large heads (≥30 mm) compared with 22-mm heads (relative risk, 1.0 vs. 3.1; P ≤ 0.001) [17]. Our study reported a dislocation rate of approximately 1.69% supporting the fact that large-diameter femoral head reduces dislocation as confirmed by several other authors [13,18-20].

In the literature, the short-term and midterm clinical results appear to be excellent, with low rates of wear, osteolysis, and aseptic loosening [13,21-26]. Plate et al. compared the effects of large-diameter (≥36 mm) and small-diameter (26 mm, 28 mm) metal femoral heads on polyethylene bearings. They reported that the large-head cohort had a mean HHS of 90 points (range: 50-100 points) and no dislocations or radiographic evidence of stem or cup loosening at a mean follow-up of five years. Gagala et al. at 3.5 years of follow-up reported excellent clinical and radiographic outcomes in 50 hips with 36-mm femoral heads. The mean HHS was 94 points, and there was no evidence of liner fractures, aseptic loosening, or osteolysis [27]. We reported average HHS of 91 (range: 85-90 points) at the final follow-up, and no case of osteolysis or loosening of cup or stem was encountered in our study.

Recently, it was postulated that larger heads generate larger frictional torques, which can contribute to corrosion and increase wear with large-head femoral diameters. A study by Dyrkacz et al. on the influence of head size on corrosion showed high corrosion scores with 36-mm bearings compared to 28-mm bearings [28]. However, no study in the literature has shown that increasing head size affects the incidence of adverse local tissue reaction (ALTR) in patients who had metal-on-polyethylene bearings. Furthermore, most cases of ALTR with metal-on-polyethylene bearings are reported in patients with smaller femoral heads (28 or 32 mm). Thus, factors other than femoral head size may be involved in the progression of ALTR [29]. Fortunately, we did not encounter any case of ALTR in our cohort.

Our results differ from studies that reported no difference in dislocation rates with head size, or those that reported a difference in functional outcomes due to head size. Ziljstra et al. reported no dislocation in a randomized control study of 50 patients who were implanted with either 28-mm heads or large-diameter metal-on-metal heads (44-66 mm). Moreover, at one-year follow-up, the mean HHS was comparable between the two groups (86 vs. 88 points) [30]. However, their study aimed to detect differences in dislocation rate rather than the functional outcome scores within the two groups.

**Table 2:** Table showing the Harris Hip Score at the last follow-up HHS, Harris Hip Score

HHS	No. of patients	Percentage
Excellent	33	55.93%
Good	26	44.07%
Fair	0	0
Poor	0	0
Total	59	100%

Our study had a few limitations. Because of the paucity of resources and financial constraints, the volumetric wear of metal-on-polyethylene implant could not be measured. Second, a lack of comparison group to compare the results of smaller versus large-head diameter femoral heads. However, the results of this study are promising and could open new research ideas for the use of large-diameter femoral head in patients requiring THR.

## Conclusions

THR using large-diameter femoral heads has become increasingly popular because of the advancements in the material properties and wear characteristics. Although the basic prerequisite for reduced dislocations is an appropriate acetabular inclination and surgical technique, the use of large-diameter femoral head provides an added advantage with a better range of motion.
